# Geographic disparities in access to outpatient stroke rehabilitation in Texas

**DOI:** 10.1371/journal.pone.0328267

**Published:** 2025-08-12

**Authors:** Joseph Wozny, Melissa Howard, Susan Doherty, Yenan Zhu, Kalyani Sonawane, Dorothea Parker, Lisa W. Thomas, Bukola Azeez, Ndi Chukwumerije, Susan Varghese, Nicholas Hoang, Suja S. Rajan, Charles Green, Vahed Maroufy, Trudy Millard Krause, Sean I. Savitz

**Affiliations:** 1 Center for Health Care Data, UTHealth, School of Public Health, Houston, Texas, United States of America; 2 Department of Neurology and Institute for Stroke and Cerebrovascular Disease, McGovern Medical School, UTHealth, Houston, Texas, United States of America; 3 Department of Management, Policy and Community Health, School of Public Health, UTHealth, Houston, Texas, United States of America; 4 Department of Graduate Studies, Cizik School of Nursing, UTHealth, Houston, Texas, United States of America; 5 Center for Evidence-Based Medicine, McGovern Medical School, UTHealth, Houston, Texas, United States of America; 6 Department of Biostatistics and Data Science, School of Public Health, UTHealth, Houston, Texas, United States of America; PLOS, UNITED KINGDOM OF GREAT BRITAIN AND NORTHERN IRELAND

## Abstract

**Background:**

Outpatient rehabilitation plays a vital role in providing post-discharge care for stroke survivors’ optimum recovery. Geographic variability in access to post-stroke rehabilitation care in rural areas is poorly understood.

**Methods:**

This study used Medicare claims from 2016 to 2019 to estimate incidence of stroke discharges home and compared rehabilitation utilization rates after discharge from acute hospitalization in the state of Texas. We also examined spatial accessibility to post-discharge outpatient rehabilitation centers between rural and urban areas. We supplemented claims results with a survey to better understand locations where outpatient rehabilitation clinics provided services to stroke patients.

**Results:**

After discharge from the hospital, patients from rural counties neighboring urban counties had lower adjusted predicted probabilities of using outpatient clinic services compared to urban areas. Patients with primary diagnosis codes of stroke sequelae: adjusted relative rate of 0.84 (CI: 0.76,0.93) with an adjusted rate difference of −0.05 (CI: −0.08, −0.02), cerebral infarction: adjusted relative rate of 0.82 (CI: 0.72,0.91) with adjusted rate difference of −0.04 (CI: −0.06,-0.02), hemorrhagic patients: adjusted relative rate of 0.81 (CI: 0.71,0.91) with an adjusted rate difference of −0.04 (CI: −0.06,-0.01). We did not find discernable differences between rural and urban areas for home health utilization or the combination of outpatient clinic services with home health as a single category. Estimates from a floating-catchment spatial accessibility model scaled from 0 (worst access) to 1 (best access) showed that, compared to urban counties, indices in rural not adjacent to urban counties were −0.16 (CI: −0.23, −0.08) lower and −0.14 (CI: −0.20, −0.08) lower in rural areas in counties adjacent to urban counties.

**Conclusions:**

Compared to urban areas, rural areas have lower spatial access to and utilization of outpatient clinic services in the state of Texas.

## Introduction

The prevalence of those living with the effects of stroke has increased as the population is growing and aging [1]. Consequently, there is an increasing demand for rehabilitation services [2]. As people discharge earlier from acute care to home, the demand for outpatient rehabilitation grows further. The greatest gains in motor recovery occur in the first 3 months post-stroke and there is a critical window for patients to receive continued outpatient therapy after acute care discharge in order to maximize outcomes [3]. The majority of stroke survivors have motor and/or cognitive impairments which impact activities of daily living (ADLs) and pose increased risks for falls, contractures, and hospital readmission [1]. A study found that patients who had contact with an outpatient therapist in the first 30 days after discharge home were less likely to be re-hospitalized in the subsequent 30 days, relative to patients who did not receive therapy [1].

For patients who do not immediately transfer from acute hospitalization to inpatient rehabilitation and are discharged home, there are a variety of options, including therapy sessions within an Outpatient Rehabilitation Site(s) (ORS), which may be a clinic or therapy center, or in the home, through a home health referral. Outpatient rehabilitation therapy is utilized to enhance recovery for many individuals across their life span [4–5]. However, access of stroke survivors to outpatient rehabilitation is poorly understood, particularly in rural versus urban environments. The lack of information on access to rehabilitation services stands in stark contrast to the extensive studies on access to acute stroke care [6].

In this study, our aim was to analyze spatial and realized access of stroke survivors to outpatient rehabilitation services in Texas, a region of the US where 70% of its counties are rural [7]. Our study is observational and descriptive in nature. Spatial access refers to the geographic availability of care locations and the time and distance involved in travelling while realized access involves patterns in utilization to determine if certain populations are not using services at the same rate as others. Our analyses revealed disparities in access and utilization across Texas, particularly in rural vs urban areas.

## Methods

### Claims data

We used deidentified Texas Medicare claims from 2016–2019 to estimate the incidence of stroke discharges home (discharge code 01: discharged home; 07: left against medical advice) and the rates of rehabilitation utilization after discharge. The study (HSC-SPH-21–0796) was approved by the institutional review board of The University of Texas Health Science Center at Houston with a waiver of authorization for informed consent based on exempt status according to 45 CFR46.104(d), with the de-identification of personal health information from the data, and the retrospective observational study design.

We identified cases using ICD-10 diagnosis codes (I60.x, I61.x, I63.x, I6789, I69.x) for stroke as the primary diagnosis on the claim. Patients had to be continuously enrolled in both Medicare Part A and B for 90 days following the discharge date from acute hospitalization. We eliminated cases if the patient had another inpatient, skilled nursing, or hospice claim within 90 days. We followed each case for 90 days, capturing all home-health visits and visits at outpatient rehabilitation sites (ORS); this includes therapy available outside the home at either rehabilitation centers or clinics in the state of Texas. We identified outpatient rehabilitation visits, which include both home health and ORS visits, using revenue codes for institutional claims and HCPCS/CPT procedure codes for professional claims. Patients were assigned to a county based on the zip code in their eligibility file at the time of discharge.

### Survey

We administered a provider survey to assess which clinics offered rehabilitation services to stroke survivors and what services they offered. We called potential centers/clinics in 240 Texas counties. Fourteen very large metropolitan counties were excluded because we focused on rural areas. We identified potential ORS by searching on Google Maps, the Medicare Provider website, and the Health Resources & Services Administration Federally Qualified Health Centers website. Willing respondents answered a voluntary survey, asking:

- If they saw adult stroke patients
  - If so, what services they offered: occupational therapy (OT), physical therapy (PT), or speech-language pathology (SLP)
  - If they were currently using tele rehab or telehealth visits for therapy
- If they did not see adult stroke patients, if they referred patients elsewhere

Out of the 1,092 potential ORSs called, 12% (131) were non-responders. 754 (69%) respondents were clearly identifiable as outpatient rehabilitation clinics/centers. Those not clearly identifiable were later determined to be entities such as staffing agencies, nursing centers, and individual practitioners not working in an ORS. Additionally, 556 (73%) of outpatient clinics/centers said they provided clinical services to adult stroke patients. We recorded survey responses in a secure spreadsheet and did not record the personal names of any respondents.

### Geographic classification

We categorized counties using the U.S. Department of Agriculture’s Urban Influence Codes (UIC), which health researchers have found useful when looking at access to care. UIC are a county level ordinal measure ranking counties from 1–12, 1 being the least rural and 12 the most. Counties’ UIC rank is defined by their adjacency to an urban/metropolitan area. Adjacency and non-adjacency are defined by a shared border. The ranks are as follows (1–2) metropolitan areas, (3–7) non-metropolitan and adjacent to a metropolitan area, and (8–12) non-metropolitan and not adjacent to a metropolitan area. Because UIC are ranked according to the most likely place for a person to live, as opposed to only the population, they can better reflect general trends for a county’s health care use and accessibility than other factors [8].

### Data processing

Claims data extraction and wrangling were done in the PostgreSQL data warehouse at the UTHealth School of Public Health Center for Healthcare Data (UTSPH CHCD). Clinics from the survey and those identified from the claims were geocoded using the TIGER geocoder PostGIS extension from each location’s publicly available address or address in the CMS National Plan and Provider Enumeration System (NPPES). To eliminate duplicates, we manually reviewed the clinic list and removed any clinics with the same name. We visually inspected clinics close to each other to ensure they had different names. Travel distance and time were calculated using pgRouting, which uses Dijkstra algorithm and OpenStreetMap data to estimate time and distance costs between locations. We used population-weighted zip code centroids as a proxy for patient location.

### Spatial accessibility calculations

To measure spatial accessibility, we used the common enhanced two-step-floating catchment area model (E2SFCA) [9]. Floating catchment models create provider to population ratios (PPR) within a travel-based limit, as opposed to a standard administrative border. Floating catchment models sum the PPR to create a spatial accessibility index (SAI) for a given populations. E2SFCA models incorporate weights to try and capture the general tendency for people to prefer small travel distances to large distances, as opposed to standard 2SFCA models, which include hard cutoffs within the catchment area and weight all locations equally. We chose the E2SFCA model because of these benefits and the fact that it’s an established model, readily available within software packages. In this paper, spatial accessibility is just one component of a broader analysis. Fully exploring more complex models of spatial accessibility would necessitate a separate, dedicated research investigation. Details of the calculations are available in the Methods of S1 Text. We also aggregated those spatial accessibility values to the county level by taking the average of the census tract values within each county. Because our population and providers are very specific, there is no established measure of accessibility to reference, like standard primary care provider ratios. Because of this, for the purposes of display, we normalized the values using min max normalization to a value from 0 to 1, 0 representing poor access and 1 good access. Spatial accessibility measures were computed using Access, a geospatial python library.

### Statistical analysis

We used a mixed-effect Poisson regression to estimate the incidence rate ratio for discharges home, which includes a random intercept at the public health region level. Because low population areas often have low stroke event counts, incidence rates may be unstable, and mapping individual county rates provides little information. To avoid that problem, we mapped a geographically smoothed rate with a geographic empirical Bayes (GEB) estimator. The GEB estimator uses observations from neighboring counties to obtain parameters that can shrink unstable rates toward the mean, without relying on a global average from all counties, as a typical EB estimator does [10]. To compare utilization rates for patients in the claims data, we used mixed-effect logistic regression. We ran a separate model for each of three binary outcomes: 1) any rehabilitation follow-up within 90 days of discharge, 2) home health follow-up within 90 days of discharge, and 3) ORS follow-up within 90 days of discharge. The exposure variable of interest was rural status of residence (urban county, rural county adjacent to urban county, rural county not adjacent to urban county). We included the following covariates: stroke type (ischemic, hemorrhagic, or stroke sequela), age (continuous), sex (male or female), and number of comorbidities prior to discharge using the Elixhauser index (continuous).Given the hierarchical structure of observations, with patients nested within counties and counties nested within public health regions, we included random intercepts at both the county and public health region levels. This approach accounts for unobserved heterogeneity and acknowledges that responses within the same county and region are correlated due to shared unobserved factors. We assumed that the variability in follow-up rates across regions and counties follows a normal distribution.For ease of interpretation, we present our results using the following quantities of interest: the average adjusted predicted probability (AAP) of follow-up, the ratio of the AAP for rural areas (relative comparison to urban areas), and the difference in AAP (absolute comparison to urban areas). Average adjusted probabilities and comparisons between them are derived by constructing a new dataset with each original categorical regressor value, fixing continuous variables at their mean values, generating predictions, and averaging the predictions over the exposure of interest. Confidence intervals for ratios and differences are derived using the delta method. The geographical empirical Bayes smoothing for incidence was calculated using GeoDa. To provide a statistical summary comparison of the indices, we used a mixed-effect linear regression with random intercepts for county and public health region, again to account for the clustering of observations, in this case, census tracts. All regression modeling was done in R. Maps were produced in QGIS.

## Results

### Incidence of stroke discharges

**Fig 1:**
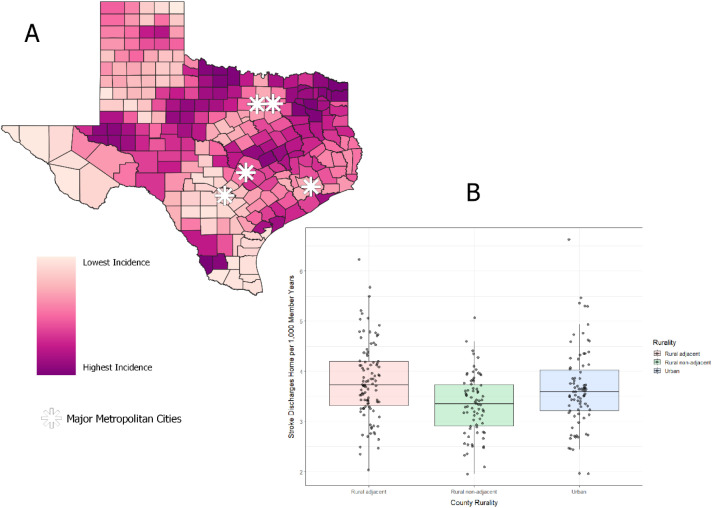
Incidence of hospital stroke discharges home. Panel A: county map of smoothed incidence of stroke discharges home per 1,000 member years. The asterisks represent the five major metropolitan areas of Texas: Houston, Austin, San Antonio, Dallas, and Forth Worth. Panel B: box plot of smoothed incidence rates.

From 2016 through 2019, there were 40,552 hospital facility claims indicating a discharge home with stroke as the primary diagnosis among Medicare beneficiaries with part A and B coverage at the time of discharge. Compared to urban counties, the incidence rate ratio for rural adjacent counties was.99 (CI:.96, 1.02) and.93 (CI:.88,.97) for rural non-adjacent counties. Fig 1A shows the geographically smoothed incidence rate by county. We can see areas with high incidence in South Texas (around Laredo), Central Texas (Houston, Austin, and Dallas which includes Waco and Temple), the region between the panhandle from the rest of Texas which stretches across Odessa/Midland, San Angelo, Abilene, and Wichita Falls. Fig 1B shows the distribution of the smoothed county rates. The median smoothed incidence rate per 1,000 member years was 3.73 (IQR: 3.31, 4.20) for rural adjacent counties, 3.35 (IQR: 2.91, 3.74) in rural non-adjacent counties, and 3.59 (IQR: 3.22, 4.03) in urban counties.

### Outpatient rehabilitation site (ORS) survey

Given the number of discharges home, we then explored rehabilitation access at ORS. Our survey revealed 774 ORS, out of which 556 (73%) provided services to stroke patients. Out of those, 359 (65%) were in urban areas, 131 (24%) in rural adjacent areas, and 63 (11%) in rural non-adjacent areas. Among the types of services provided, 539 sites (97%) offered PT, 248 (45%) offered OT, and 192 (35%) offered SLP; 160 sites (29%) offered all three services, and 238 (30%) provided TR. For rural non-adjacent clinics, 36% offered OT, 95% PT, and 37% SLP, and 30% all three services; for rural adjacent areas, 49% offered OT, 97% PT, and 40% SLP, and 36% all three services; for urban areas, 42% offered OT, 95% PT, 30% SLP, and 26% all three services.

**Fig 2:**
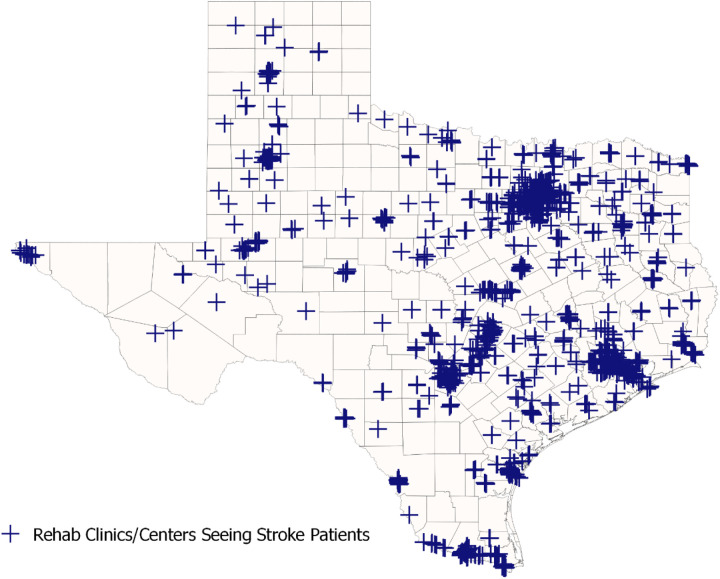
Clinics seeing stroke patients in Texas. The map includes all clinics in the survey that reported seeing stroke patients as well as all clinics with rehabilitation claim featuring a stroke diagnosis in 2019.

**Fig 3:**
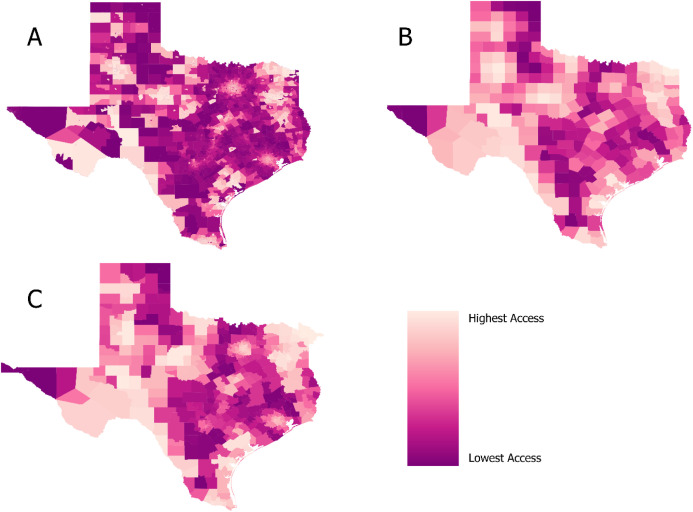
Enhanced 2-step floating catchment area spatial accessibility index at different geographic levels. Panel A: E2SFCA indices at 30 minutes aggregated by census tract; Panel B: tracts averaged at the county level; Panel C: tracts averaged by Dartmouth Atlas’s Primary Care Service areas.

**Fig 4:**
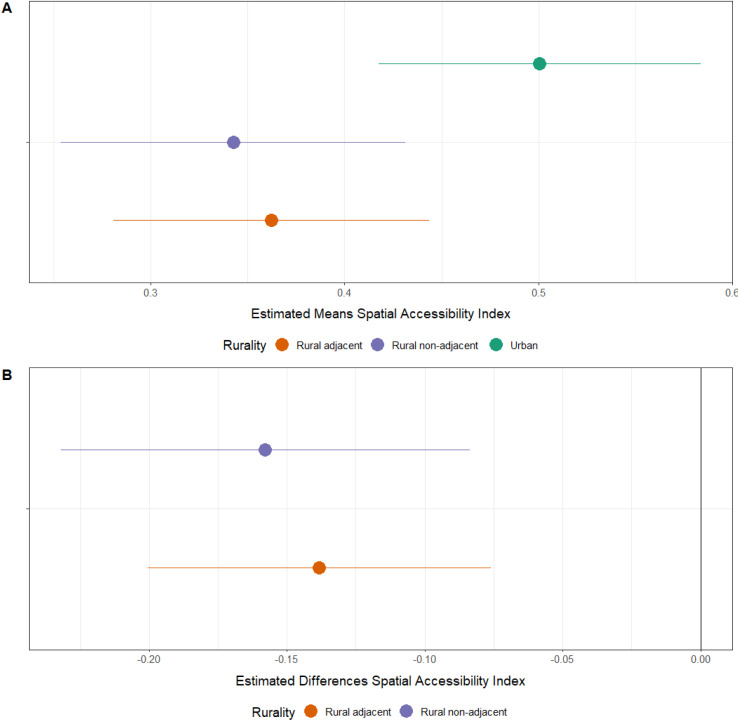
Estimates for means and differences in spatial accessibility indices for tracts by county type. Panel A: estimated means for tract SAI by county type. Panel B: estimated differences between areas in rural and urban counties. Both mixed-effect linear models include random intercept terms for public health region and county.

Fig 2 shows the locations of all ORS derived from our provider survey and those from the claims data used for our spatial analysis. Fig 3A then shows the spatial accessibility model of ORS at the census tract level. Predictably, urban centers like San Antonio, Houston, and Dallas have high accessibility while accessibility drops outside the city. There is also higher accessibility between the cities that follows the path of the major highways: I35 from San Antonio through Austin to Dallas and I45 from Houston to Dallas. Because of the structure of tracts, several areas appear dark purple (lowest access) but have high accessibility areas in their centers, reflecting small towns within sparsely populated regions. Some apparent discrepancies are explained here. For example, although Fig 2 shows the ORS sites in West Texas around El Paso, the area still shows low accessibility in Fig 4 due to the large population that can access the small number of outpatient clinics/centers. Those patients may be traveling into neighboring areas outside Texas for providers because the area to the east is largely uninhabited. Fig 3B shows the average accessibility values aggregated by county, and Fig 3C shows the Dartmouth Atlas Primary Care Service Areas (PCSA). Fig 4A shows the adjusted average mean spatial accessibility indices after adjusting for geographic clustering within counties and public health regions. Values have been normalized with 0 as the lowest spatial accessibility and 1 as the highest. Fig 4B shows the estimated differences from the same model. On average, compared to census tracts in urban areas, the estimated spatial accessibility indices for areas in rural counties not adjacent to urban counties were −0.16 (CI: −0.23, −0.08) lower and −0.14 (CI: −0.20, −0.08) lower for areas in rural counties adjacent to urban counties.

### Outpatient rehabilitation utilization

We identified 27,243 discharges home meeting the inclusion criteria and followed them for 90 days after discharge. Table 1 provides details. 22,373 cases of new discharges had an enrollment zip code in an urban county (82%), 3,549 (13%) in a rural adjacent county, and 1,321 (5%) in a rural non-adjacent county. Out of the 27,243 stroke discharges to the home setting, 16,855 (62%) had an ORS or home health claim within 90 days of discharge: 7,179 (26%) had an ORS claim and 11,205 (41%) had a home health claim.

**Table 1:** Patient characteristics for utilization analyses.

Characteristic	Urban, N = 22,373^*^	Rural adjacent, N = 3,549^*^	Rural non-adjacent, N = 1,321^*^	p-value^†^
Age	75 (69, 82)	75 (69, 81)	74 (69, 81)	<0.001
Sex				0.001
Female	10,999 (49%)	1,648 (46%)	606 (46%)	
Male	11,374 (51%)	1,901 (54%)	715 (54%)	
Diagnosis				<0.001
Stroke Sequelae	9,517 (43%)	1,373 (39%)	543 (41%)	
Cerebral Infarction	11,912 (53%)	2,018 (57%)	729 (55%)	
Hemorrhagic	944 (4.2%)	158 (4.5%)	49 (3.7%)	
Elixhauser Comorbidities	8 (5, 11)	7 (4, 11)	7 (4, 11)	<0.001
Center/Clinic rehabilitation visit within 90 days	6,110 (27%)	742 (21%)	327 (25%)	<0.001
Home Health rehabilitation visit within 90 days	9,173 (41%)	1,514 (43%)	518 (39%)	0.061
Any rehabilitation visit within 90 days	13,988 (63%)	2,084 (59%)	783 (59%)	<0.001

* Median (IQR); n (%)

† Kruskal-Wallis rank sum test; Pearson’s Chi-squared test

**Fig 5:**
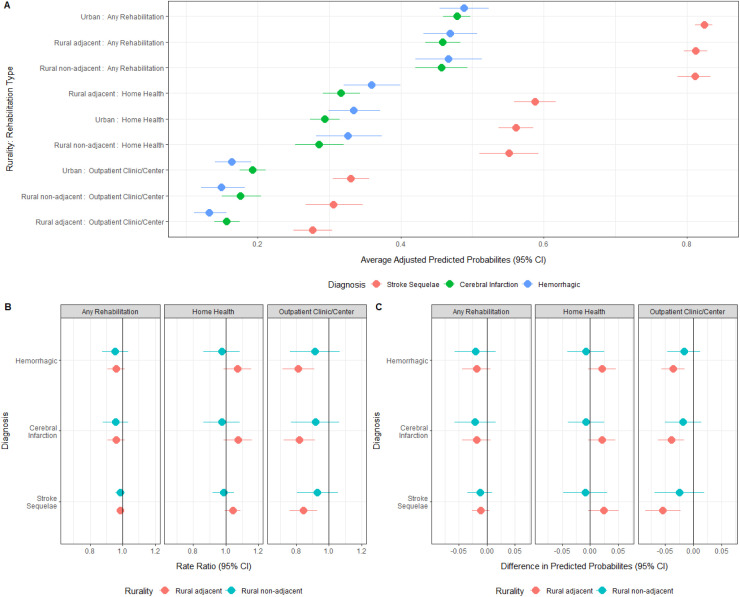
Rehabilitation utilization. Panel A: Average Adjusted Probabilities. Panel B: Rate Ratios for Average Adjusted Probabilities. Panel C: Absolute Differences in Average Adjusted Probabilities. All quantities of interest were generated by taking predictions from a mixed-effect logistic regression with continuous values fixed at their mean value.

Fig 5A shows the average adjusted predicted probabilities (AAP) for follow-up. For all outcomes, patients discharged with a diagnosis of stroke sequelae had the highest AAP of follow-up, with cerebral infarction and hemorrhagic strokes lagging. The AAP for home health utilization was higher than ORS settings. Fig 5B displays the adjusted relative ratio in probabilities for rural areas and Fig 5C shows the adjusted absolute difference in probabilities. With the current sample size, we were not able to find discernable evidence for differences between rural non-adjacent and urban counties in any rehabilitation category. For ORS follow-up in rural adjacent areas, our data showed lower relative rates for all stroke types: 0.81 (hemorrhagic; CI: 0.71,0.91), 0.82 (cerebral Infarction; CI: 0.72–0.91), and 0.84 (stroke sequelae; CI: 0.76,0.93). In absolute terms, the differences in AAP were −0.04 (hemorrhagic; CI: −0.06, −0.01), −0.04 (cerebral infarction; CI: −0.06, −0.02), and −0.05 (stroke sequelae; CI: −0.08, −0.02). Although point estimates were higher for home health utilization in rural adjacent areas compared to urban areas, our data did not provide sufficient evidence for a clear difference. Regarding any follow-up, including home health or ORS, our sample did not show discernable evidence that rates differed between urban and rural counties. A full table of all numerical values are provided in Tables A, B, and C in S1 Text.

### Additional data on outpatient rehabilitation visits

For those patients who did receive ORS care, the median travel time was 23 minutes (IQR: 11, 52) in urban counties, 52 minutes (IQR: 13, 105) in rural non-adjacent counties, and 30 minutes (IQR: 5, 115) in rural adjacent counties. The median number of visits, measured by distinct days with a rehab visit, within 90 days were similar for all county types, between 9 and 10 for ORS visits, between 15 and 16 for home health, and 15 for any type of visit. The median number of days before first rehabilitation visit was 5 days for all county types.

## Discussion

Access to care has been well-studied for patients with acute ischemic stroke [6]. Several studies have documented increased access of the patient population over the years to stroke centers for acute stroke management and reperfusion therapy. Post-acute stroke care involving rehabilitation has not received the same level of investment in order to maintain the benefits gained by early diagnosis and acute treatment [11]. Rehabilitation care has far-reaching effects to facilitate recovery and transition to the community, with studies showing the earlier rehab therapy begins, the better the outcomes [8–10]. In the post-acute setting, a few studies have begun examining the inequities among stroke survivors to access inpatient rehabilitation [12]. Far less is known about access to outpatient rehabilitation in the months after hospitalization. In rural areas, stroke patients were more likely to use home health services than patients in urban areas and less likely to visit outpatient centers or have any outpatient services within 90 days.

As we assumed, average spatial accessibility to outpatient rehabilitation sites is lower in rural areas. However, the distribution of accessibility across rural counties varies. Compared to rural counties in West Texas, rural counties between the major metropolitan cities seem to have lower accessibility. More rural Texans live in these counties than the rural counties in West Texas. It is good to be cautious before jumping to conclusions that a county lacks access because there are fewer providers within its borders. There may be little demand for providers, or there may be providers across a county border. Because the E2SFCA does not use hard cut-off boundaries like population to provider ratio (PPR), we encountered interesting situations. Although patients may access more providers according to the model’s drive time area, their accessibility may not increase because other people also have increased access to that provider. They effectively end up with more access to busier providers. On the other hand, areas with only a few providers may have little demand and lead to a higher spatial accessibility index for an area despite a low number of providers. Aggregating the values from the E2SFCA back to a lower resolution scale at the county and PCSA level seemed to present a balanced view of variation in the state with the benefit of interpretability to the general reader familiar with county boundaries. This approach presents an alternative to using county boundaries as the initial unit of aggregation via counting, as many do, which may make the questionable assumption that county boundaries are a realistic representation of utilization patterns.

Our data showed rural stroke survivors sometimes face long commutes in order to visit outpatient rehabilitation centers in urban areas. Many of these trips were well above one hour. It is a limitation that enrollment zip in claims data is not a certain indication from where a patient is travelling, in any given point of the year. Our survey is in line with nationwide findings, showing that while 70% of urban U.S. citizens are within a 30-minute drive of a stroke center, which often includes outpatient services, this is true of only 26% of rural residents [13].

Rural areas not only face a shortage of outpatient rehabilitation services but the types of services offered at outpatient sites is also concerning. There were quite limited services provided that address the different disciplines of neurorehabilitation required to treat the whole patient after a stroke. The number of ORCs offering PT was more than double that offering OT, a service specializing in return to function with ADLs. Speech therapy was even lower than OT, offered by only 25% of sites responding to our ORS survey.

The lack of outpatient rehabilitation centers led us to pursue the question whether rural patients might receive more home health services, but the data did not support that hypothesis. Our data did not support a higher frequency of home health rehabilitation visits between rural and urban areas, but the median number of visits was noticeably higher for home health compared to ORS. There is scant and conflicting published information on the duration, quality, or effectiveness of home-based therapy compared with outpatient rehabilitation facility therapy [14]. Provider credential information in the NPPES file is not standardized and the provider identifier is often missing on the home health facility claims, making it difficult to assess the expertise of the provider.

Given the limited availability and high travel times for rural patients to access outpatient rehabilitation centers, and the difficulty of making decisions about allocation based on a population that is a very small subset of all rural patients, it raises the question whether telerehabilitation could enhance health services in underserved areas. However, to provide this type of service, patients in rural areas require adequate home internet bandwidth and video display technology, whether by computer of phone. A map of broadband internet access in Texas counties is displayed in S1 Text Fig A. Unfortunately, nearly 30% of the population in rural Texas does not have broadband internet and 12% do not have a computer but do have a cell phone. Even in metropolitan areas, 20% of the urban population does not have broadband internet in Texas. Some areas where ORS access estimates were low also have low internet access, such as South Texas between Laredo and San Antonio, Central Texas between Houston and other metropolitan areas, and in the Panhandle, between Wichita Falls, Laredo, Amarillo. Thus, the same regions with high incidence of stroke and poor access to rehabilitation centers happen to be the same places where there is less internet.

This problem speaks to the importance of policy decisions concerning infrastructure as a key part of the actions needed to improve rural access to care. The pandemic spurred adoption of telehealth for a range of medical conditions [15]. A national database review found 69% of clinics in 49 states implemented telerehabilitation for physical therapy services during the pandemic [16].

One randomized trial suggested that telerehabilitation provides equal treatment effects of the arm compared with in-person therapy [14]. Along with the need for infrastructure changes, we need additional randomized trials on the effectiveness of telerehabilitation and which types of therapy will be most appropriate for different types of impairments, using telemedicine [16].

There are several limitations to our study. Results from Medicare data may not generalize the experience of younger stroke patients, who would be captured in a commercial claims dataset. Claims are insurance generated data; they contain limited personal and clinical data. The sample size for rural non-adjacent counties was small, increasing the width of the confidence intervals. Claims only capture insured individuals; Texas is the state with the largest percentage of uninsured individuals under 65, and rural areas on average have a higher percentage of uninsured persons. These individuals who are not captured in claims are the ones who may suffer the most from a lack of access to healthcare. By only using Texas data, we assume we missed some cross-state utilization, which casts uncertainty on areas near borders. The unique population and geography of Texas, such as it having the highest number of counties, may mean that these results do not easily generalize to other states that have different demographic features and geographic structure. Using counties as the unit of analysis has its drawbacks. Texas is a large state with the highest number of counties. By specifying discharges home, rather than just stroke occurrence, there were very few observations in some rural areas, even over the course of multiple years. Geographic access, captured by floating catchment models, is only part of the picture. There are other barriers to care, such as socioeconomic factors, that a model based on travel alone does not capture. There were additional limitations in our survey: the 12% non-responder rate may introduce bias into the results. Our analysis also focuses only on the availability of care and does not capture the quality of care or future outcomes of these patients, which is a topic for further research.

## Conclusions

For those patients who would benefit from services provided by an outpatient rehabilitation site, we find a discernable geographic disparity in utilization. Until internet coverage is provided to all rural populations to facilitate telerehabilitation, we need other solutions to deliver robust therapeutic interventions in order to enhance recovery for stroke survivors. Further information is needed on quality differences between settings of post-acute care. Additional work must be done to identify whether rurality impacts access to ORS and should be considered a concerning disparity in patterns of care.

## Supporting information

S1 Text Supplementary information. Additional method details, tables, and figures referenced in text.
(DOCX)
