# Supplementary material for: Geographic disparities in access to outpatient stroke rehabilitation in Texas
Source: PLoS One. 2025 Aug 12;20(8):e0328267. doi: 10.1371/journal.pone.0328267 (PMC12342321; doi:10.1371/journal.pone.0328267)
Supplement: S1 Text — Additional method details, tables, and figures referenced in text. (DOCX) [file pone.0328267.s001.docx]

**S1 Text. Supplementary Information.**

**S1 Methods**

**Spatial Accessibility Details**

In a basic two-step floating catchment model, the first step is to get a provider to population ratio for the supply of providers. $s_{l}$ is the supply of providers at location $l$ and $d_{r}$ is the population demand at census tract location $r$ in the year 2019. The set of reachable locations $r\in T_{l}$ is all $r$ within the time buffer $T_{l}$, which is a given set of minutes or miles of location $l$.

$$R_{l}\equiv\frac{s_{l}}{\sum_{r\in T_{l}} d_{r}}$$

For each population center $r$, the ratios of all providers $R_{l}$ within the drive time radius $T_{r}$of the population location are summed.

$$2SFCA(r)\equiv\sum_{l\in T_{r}} R_{l}$$

One provider may be within the service area of several origin points, each placing demand on their services. Likewise, many different provider areas may overlap in a certain population origin—the 2SFCA sums of all the different ratios.

The E2SFCA expands on the 2SFCA by including a weighted factor to consider the fact that people in general would prefer to visit somewhere closer than further away (Luo and Qi, 2009). $W\left( t_{rl} \right)$ is an impedance function that decreases as travel time increases. Populations further away from providers place less demand on providers and providers further away from populations are less available. In our case it’s given by the gaussian decay function. The basic framework of the E2FCA ratio is the same as 2SFCA, only now in the first step a weight depending on the travel time is multiplied first to the population (denominator), producing a weighted provider to population ratio:

$$R_{l}\equiv\frac{s_{l}}{\sum_{r} d_{r}W\left( t_{rl} \right)}$$

The weight is again applied to the provider side and the ratio is summed just as before in the 2SFCA.

$$E2SFCA(r)\equiv\sum_{l} R_{l}W\left( t_{rl} \right)$$

**S1 Table A**

**Average Adjusted Predicted Probabilities (95% CI) for Rehabilitation Outcomes Compared to Urban Areas**

| Diagnosis | Rurality | Adjusted Predicted Probability | 95% CI |
| --- | --- | --- | --- |
| Any Rehabilitation | | | |
| Cerebral Infarction | Urban | 0.48 | (0.46,0.50) |
| Cerebral Infarction | Rural adjacent | 0.46 | (0.43,0.48) |
| Cerebral Infarction | Rural non-adjacent | 0.46 | (0.42,0.49) |
| Stroke Sequelae | Urban | 0.82 | (0.81,0.84) |
| Stroke Sequelae | Rural adjacent | 0.81 | (0.80,0.83) |
| Stroke Sequelae | Rural non-adjacent | 0.81 | (0.79,0.83) |
| Hemorrhagic | Urban | 0.49 | (0.45,0.52) |
| Hemorrhagic | Rural adjacent | 0.47 | (0.43,0.51) |
| Hemorrhagic | Rural non-adjacent | 0.47 | (0.42,0.51) |
| Outpatient Clinic/Center | | | |
| Cerebral Infarction | Urban | 0.19 | (0.18,0.21) |
| Cerebral Infarction | Rural adjacent | 0.16 | (0.14,0.17) |
| Cerebral Infarction | Rural non-adjacent | 0.18 | (0.15,0.21) |
| Stroke Sequelae | Urban | 0.33 | (0.31,0.36) |
| Stroke Sequelae | Rural adjacent | 0.28 | (0.25,0.30) |
| Stroke Sequelae | Rural non-adjacent | 0.31 | (0.27,0.35) |
| Hemorrhagic | Urban | 0.16 | (0.14,0.19) |
| Hemorrhagic | Rural adjacent | 0.13 | (0.11,0.16) |
| Hemorrhagic | Rural non-adjacent | 0.15 | (0.12,0.18) |
| Home Health | | | |
| Cerebral Infarction | Urban | 0.29 | (0.27,0.31) |
| Cerebral Infarction | Rural adjacent | 0.32 | (0.29,0.34) |
| Cerebral Infarction | Rural non-adjacent | 0.29 | (0.25,0.32) |
| Stroke Sequelae | Urban | 0.56 | (0.54,0.59) |
| Stroke Sequelae | Rural adjacent | 0.59 | (0.56,0.62) |
| Stroke Sequelae | Rural non-adjacent | 0.55 | (0.51,0.59) |
| Hemorrhagic | Urban | 0.33 | (0.30,0.37) |
| Hemorrhagic | Rural adjacent | 0.36 | (0.32,0.40) |
| Hemorrhagic | Rural non-adjacent | 0.33 | (0.28,0.37) |

S1 Table B

**Adjusted Rate Ratios (95% CI) for Rehabilitation Outcomes Compared to Urban Areas**

| Rurality | Diagnosis | Adjusted Rate Ratio | 95% CI |
| --- | --- | --- | --- |
| Any Rehabilitation | | | |
| Rural adjacent | Cerebral Infarction | 0.96 | (0.91,1.01) |
| Rural adjacent | Stroke Sequelae | 0.99 | (0.97,1.00) |
| Rural adjacent | Hemorrhagic | 0.96 | (0.91,1.01) |
| Rural non-adjacent | Cerebral Infarction | 0.96 | (0.88,1.03) |
| Rural non-adjacent | Stroke Sequelae | 0.99 | (0.96,1.01) |
| Rural non-adjacent | Hemorrhagic | 0.96 | (0.88,1.03) |
| Outpatient Clinic/Center | | | |
| Rural adjacent | Cerebral Infarction | 0.82 | (0.72,0.91) |
| Rural adjacent | Stroke Sequelae | 0.84 | (0.76,0.93) |
| Rural adjacent | Hemorrhagic | 0.81 | (0.71,0.91) |
| Rural non-adjacent | Cerebral Infarction | 0.92 | (0.77,1.06) |
| Rural non-adjacent | Stroke Sequelae | 0.93 | (0.80,1.05) |
| Rural non-adjacent | Hemorrhagic | 0.91 | (0.76,1.06) |
| Home Health | | | |
| Rural adjacent | Cerebral Infarction | 1.07 | (0.99,1.16) |
| Rural adjacent | Stroke Sequelae | 1.04 | (0.99,1.09) |
| Rural adjacent | Hemorrhagic | 1.07 | (0.99,1.16) |
| Rural non-adjacent | Cerebral Infarction | 0.97 | (0.86,1.09) |
| Rural non-adjacent | Stroke Sequelae | 0.98 | (0.92,1.05) |
| Rural non-adjacent | Hemorrhagic | 0.97 | (0.86,1.09) |

**S1 Table C**

**Difference in Average Adjusted Predicted Probabilities (95% CI) for Rehabilitation Outcomes Compared to Urban Areas**

| Rurality | Diagnosis | Difference | 95% CI |
| --- | --- | --- | --- |
| Any Rehabilitation | | | |
| Rural adjacent | Cerebral Infarction | -0.02 | (-0.04,0.01) |
| Rural adjacent | Stroke Sequelae | -0.01 | (-0.03,0.00) |
| Rural adjacent | Hemorrhagic | -0.02 | (-0.04,0.01) |
| Rural non-adjacent | Cerebral Infarction | -0.02 | (-0.06,0.02) |
| Rural non-adjacent | Stroke Sequelae | -0.01 | (-0.03,0.01) |
| Rural non-adjacent | Hemorrhagic | -0.02 | (-0.06,0.02) |
| Outpatient Clinic/Center | | | |
| Rural adjacent | Cerebral Infarction | -0.04 | (-0.06,-0.02) |
| Rural adjacent | Stroke Sequelae | -0.05 | (-0.08,-0.02) |
| Rural adjacent | Hemorrhagic | -0.04 | (-0.06,-0.01) |
| Rural non-adjacent | Cerebral Infarction | -0.02 | (-0.05,0.01) |
| Rural non-adjacent | Stroke Sequelae | -0.02 | (-0.07,0.02) |
| Rural non-adjacent | Hemorrhagic | -0.02 | (-0.05,0.01) |
| Home Health | | | |
| Rural adjacent | Cerebral Infarction | 0.02 | (0.00,0.04) |
| Rural adjacent | Stroke Sequelae | 0.02 | (0.00,0.05) |
| Rural adjacent | Hemorrhagic | 0.02 | (0.00,0.05) |
| Rural non-adjacent | Cerebral Infarction | -0.01 | (-0.04,0.02) |
| Rural non-adjacent | Stroke Sequelae | -0.01 | (-0.05,0.03) |
| Rural non-adjacent | Hemorrhagic | -0.01 | (-0.04,0.03) |


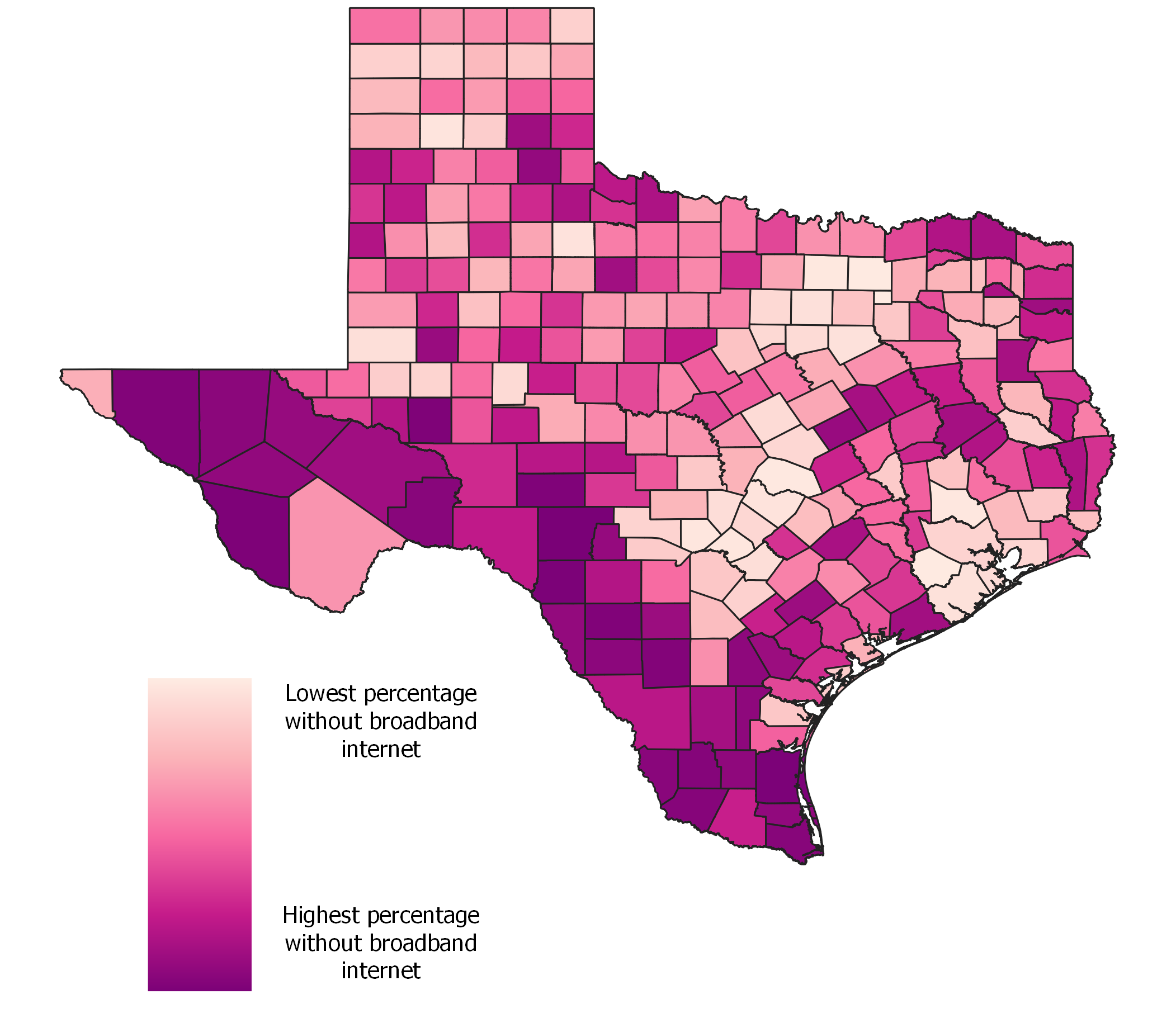


S1 Fig A

**Map of the Percentage of Texas County Population without Broadband Internet**

Data from 2019 Census Bureau 5-Year American Community Survey
